# Myxedema Coma as the Initial Presentation of Undiagnosed Hypothyroidism: A Rare but Reversible Emergency

**DOI:** 10.7759/cureus.98621

**Published:** 2025-12-07

**Authors:** Rabia Mansoor, Maheen Iqbal, Tushaar Kakkar, Aymen Bader, Taha Elsahy

**Affiliations:** 1 General Medicine, Peterborough City Hospital, Peterborough, GBR; 2 Acute Medicine, Peterborough City Hospital, Peterborough, GBR; 3 Internal Medicine, Peterborough City Hospital, Peterborough, GBR; 4 Acute Medicine, Royal Derby Hospital, Derby, GBR

**Keywords:** endocrinology, glasgow coma scale (gcs), laboratory investigations, myxedema coma, thyroid-stimulating hormone (tsh)

## Abstract

Myxedema coma is a rare, life-threatening endocrine emergency representing the extreme manifestation of untreated hypothyroidism. It usually presents with nonspecific symptoms such as confusion, hypothermia, and bradycardia, which can lead to delayed recognition, particularly in elderly patients with multiple comorbidities. We report the case of a 77-year-old man with hypertension, stage 4 chronic kidney disease, anemia of chronic disease, myeloma, and prostate cancer who presented with reduced consciousness following a period of increasing lethargy and confusion. On arrival, he was hypotensive and hypothermic with a Glasgow Coma Scale (GCS) score of 8/15 and extensive lower limb edema, extending up to the abdomen. Laboratory investigations revealed severe hypothyroidism (thyroid-stimulating hormone (TSH) >99.9 mU/L; free thyroxine (T4) 8 pmol/L) and mild infection. A diagnosis of myxedema coma was made, and he was treated with intravenous hydrocortisone, nasogastric levothyroxine (later addition of intravenous liothyronine after discussion with the endocrinology team), antibiotics, and supportive measures, including cautious rewarming and fluid resuscitation. His level of consciousness improved within 48 hours, and thyroid function gradually normalized. The case illustrates how myxedema coma may be the first manifestation of previously undiagnosed hypothyroidism and highlights the diagnostic challenge posed by overlapping comorbidities.

## Introduction

Myxedema coma, also known as myxedema crisis, is an extreme, life-threatening presentation of severe hypothyroidism and remains a medical emergency. It denotes a state of profound metabolic derangement and multisystem dysfunction in the setting of longstanding untreated or severely decompensated hypothyroidism [1]. The condition can affect any age group, but significantly higher morbidity is seen in elderly patients, and it carries a high mortality rate even with prompt treatment and diagnosis [2].

Although the eponym includes "coma", it can present with a spectrum of neurological and non-neurological symptoms. The clinical presentation is often nonspecific and may overlap with other causes of altered level of consciousness, sepsis, and multi-organ dysfunction, which contributes to diagnostic delay [3]. Features can include hypothermia, bradycardia, hypotension, hyponatremia, hypoventilation with carbon dioxide (CO₂) retention, and generalized non-pitting edema [4].

Triggers for decompensation in hypothyroid patients often include recurrent illnesses such as infection or sepsis, extreme cold exposure, sedative use, cardiovascular events, or metabolic disturbances, all of which can precipitate decompensation in already susceptible patients [5].

A systematic survey published in 2025 estimated a mortality rate of 38.8% among 698 cases of myxedema coma identified from published series over the prior 20 years. Therefore, early recognition of warning signs is crucial for improving patient outcomes [2,6].

Myxedema coma often occurs in patients with known hypothyroidism that is untreated, is undertreated, or has been subjected to medication non-compliance. Recent analyses show that many patients presenting with myxedema coma have no prior diagnosis of hypothyroidism, underscoring the need for clinical vigilance while dealing with multi-organ dysfunction, especially in the elderly [7]. Early recognition and immediate initiation of thyroid hormone replacement, corticosteroids, and supportive measures remain the cornerstones of management. This case highlights a rare presentation of previously undiagnosed hypothyroidism manifesting as myxedema coma in an elderly man with multiple comorbidities. It underscores the importance of considering thyroid dysfunction in elderly patients presenting with unexplained hypothermia, bradycardia, or altered mental status.

This case report was previously presented as a poster at the 2025 Scottish Society of Physicians Annual Meeting on September 19, 2025, and the abstract was submitted to the Scottish Medical Journal.

## Case presentation

A 77-year-old man with a background of hypertension, stage 4 chronic kidney disease, multiple myeloma, prostate cancer, and anemia of chronic disease was brought to the emergency department (ED) after being found drowsy and minimally responsive at home. According to his family, he had been increasingly lethargic and withdrawn over the preceding week, with reduced oral intake and intermittent confusion. There was no known history of thyroid disease or previous thyroid function testing. The family also reported experiencing a gradual functional decline over the past few months.

**Figure 1:**
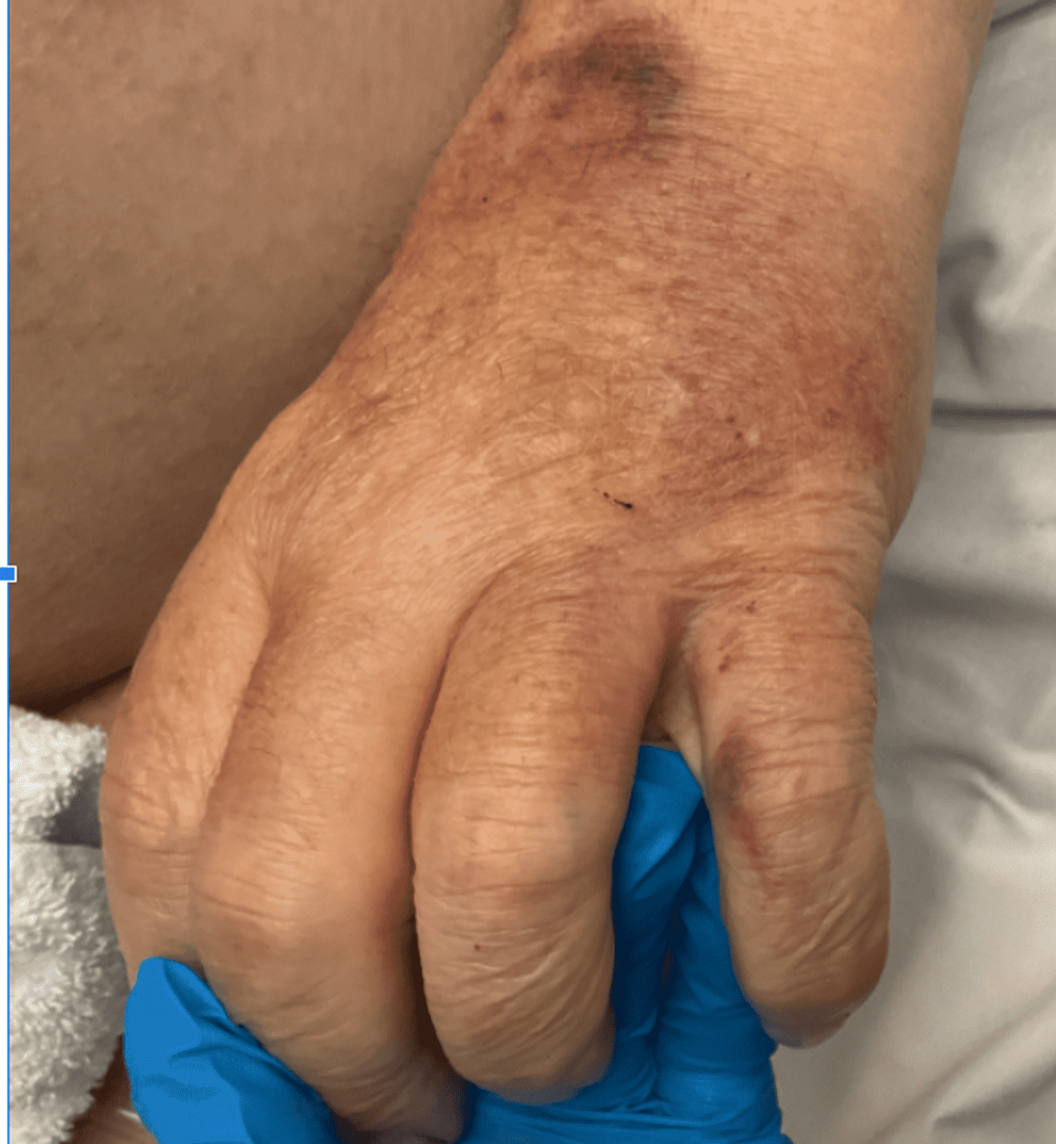
Edematous hand

On arrival, he appeared pale and cold to the touch. His vital signs were as follows: blood pressure 65/47 mmHg, heart rate 59 beats per minute, respiratory rate 10 breaths per minute, temperature 34.1°C, and oxygen saturation 93% on room air. His Glasgow Coma Scale (GCS) score was 8 out of 15 (eye response 2, verbal response 2, motor response 4). Physical examination revealed dry skin, periorbital puffiness, and anasarca (bilateral lower limb edema extending up to the abdomen and swollen hands as shown in Figure 1).

Cardiovascular and respiratory examinations were largely unremarkable, apart from bradycardia and reduced chest expansion. Neurological examination revealed no focal deficits.

**Figure 2:**
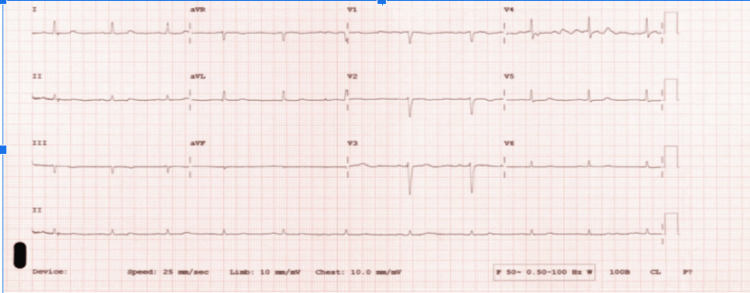
Initial electrocardiogram: sinus bradycardia with low-voltage QRS complexes

Initial laboratory investigations demonstrated severe hypothyroidism, with a thyroid-stimulating hormone (TSH) level greater than 99.9 milliunits per litre and a free thyroxine (T4) level of 8 picomoles per litre. Inflammatory markers were mildly elevated, but no clear focus of infection was identified. Urea and creatinine were raised, consistent with underlying renal impairment. The random cortisol level was within the normal range. The electrocardiogram (ECG) showed sinus bradycardia with low-voltage QRS complexes as seen in Figure 2.

Chest X-ray (CXR) revealed linear atelectasis in the lower zones bilaterally, suggestive of inflammatory changes due to infection.

A computed tomography (CT) scan of the head showed incidental opacification of the right mastoid air cells and middle ear cavity, suggesting otomastoiditis with bony erosion anteriorly into the temporomandibular joint (TMJ). The findings were discussed with the otolaryngology (ear, nose, and throat (ENT)) team, who advised no further intervention at that stage.

Given his presentation and biochemical findings, a diagnosis of myxedema coma was made. Differential diagnoses at admission included acute confusion of unknown origin and multi-organ failure. However, the combination of hypothermia, bradycardia, and markedly deranged thyroid function confirmed the diagnosis.

Empirical broad-spectrum antibiotics were initiated while awaiting culture results. Intravenous hydrocortisone 100 milligrams every eight hours was started to prevent adrenal insufficiency, followed by nasogastric levothyroxine 300 micrograms STAT and then 100 micrograms once daily. As per discussion with the endocrinology team, intravenous liothyronine (5-20 micrograms once daily) was later added to augment thyroid hormone replacement. Supportive management included cautious rewarming with a warm air blanket, intravenous fluid resuscitation, and continuous cardiac monitoring. Over the next 48 hours, his mental status gradually improved; he became more alert, responsive, and able to follow simple commands. Repeat thyroid function tests showed a gradual rise in free T4 and an early decline in TSH levels (shown in Table 1).

**Table 1:** Thyroid hormone levels showing gradual rise in free T4 and early decline in TSH levels TSH: thyroid-stimulating hormone; T4: thyroxine

	Reference range	08/01/2025	08/01/2025	27/01/2025	11/03/2025
TSH	0.30-4.20 units: mU/L	>99.9	>99.9	30.0	56.0
Free T4	11.9-21.6 units: pmol/L	8.0	8.4	12.4	11.9

During his hospital stay, he developed an episode of hospital-acquired pneumonia (HAP), which was treated successfully with targeted antibiotics. His renal function temporarily worsened, necessitating emergency dialysis. He also developed hematuria requiring bladder irrigation. No cardiac arrhythmias were recorded during his admission.

Over the following weeks, his biochemical profile continued to improve, and his GCS score returned to baseline. After approximately 50 days in the hospital, he was medically stable and transferred to a rehabilitation facility for ongoing functional recovery. Repeat thyroid function tests at the time of discharge to the rehabilitation center showed a TSH level of 56 milliunits per litre with rising free T4 levels.

During rehabilitation, while awaiting his package of care, he developed vomiting after consuming custard, which led to aspiration pneumonia. Unfortunately, despite prompt medical attention, he passed away.

## Discussion

Myxedema coma is often a difficult diagnosis to establish, particularly when it represents the first manifestation of hypothyroidism in elderly patients. It can occur as the initial presentation of any underlying cause of hypothyroidism, whether autoimmune, iatrogenic, or idiopathic [8].

Myxedema coma is characterized by a range of systemic symptoms, with neurological dysfunction being among the most common clinical manifestations. The pathophysiology and diverse presentations of this condition reflect the extensive physiological influence of thyroid hormone. These hormones influence virtually all cells in the body by activating or repressing a variety of genes after binding to thyroid hormone receptors [9]. As a result, a deficiency of thyroid hormone disrupts metabolic regulation and organ function on multiple levels. Hypothermia and reduced respiratory drive contribute to peripheral vasoconstriction, which in turn compromises thermoregulation and precipitates hypotension. Cardiovascular involvement is common and may include hypotension, cardiogenic shock, arrhythmias, and varying degrees of heart block [9]. Neurologically, patients may present with lethargy, confusion, or reduced consciousness. Other frequently described features include depression, psychosis, disorientation, delayed reflexes, paranoia, and memory impairment [10]. Respiratory depression, abdominal pain, nausea, vomiting, electrolyte disturbances such as hyponatremia, and an increased tendency to bleed are also recognized components of the clinical picture.

The case being discussed in this report presented with disorientation, reduced GCS score, hypotension, and hypothermia. His extensive comorbidities, including chronic kidney disease, myeloma, and malignancy, could each have contributed partially to his symptoms but did not fully explain the presentation. The classical myxedematous features observed on examination prompted the evaluation of thyroid function, which confirmed the diagnosis.

Given its rarity, estimated at approximately 0.22 cases per million people annually in the Western world [11], and its broad, often nonspecific presentation, large-scale prospective studies on myxedema coma remain impractical [4]. To overcome this limitation, a 2014 retrospective study proposed a scoring system to facilitate early diagnosis [12]. Scores greater than 60 were found to be highly suggestive of myxedema coma, while values above 45 identified patients at increased risk. The scoring criteria included cardiovascular, neurological, gastrointestinal, and metabolic parameters, providing a structured clinical framework that can support timely diagnosis in ambiguous cases.

The optimal initial thyroid hormone replacement in myxedema coma remains uncertain. Liothyronine acts faster but carries a higher risk of cardiac complications, so it is generally added later to levothyroxine. More evidence is needed to determine if combination therapy improves survival, especially in elderly patients.

## Conclusions

Using this case, we would like to highlight the diagnostic dilemma posed by myxedema coma, particularly when it presents as the initial manifestation of undiagnosed hypothyroidism.

Men are four times less likely to present with hypothyroidism, and the presentation can easily be mistaken for infection, metabolic encephalopathy, or any other cause of reduced consciousness. Clinicians should maintain a high index of suspicion for hypothyroidism in the list of differentials in patients who present with generalized multisystemic shutdown, especially with infection acting as a trigger, and perform relevant examinations and send appropriate tests, such as thyroid function tests.

To conclude, despite the high mortality associated with this condition, meaningful recovery is achievable with early recognition, timely initiation of corticosteroids and thyroid hormone replacement, and meticulous supportive care coordinated through a multidisciplinary approach.
